# Supplementary material for: Discovery of Sulfated Small Molecule Inhibitors of Matrix Metalloproteinase-8
Source: Biomolecules. 2020 Aug 9;10(8):1166. doi: 10.3390/biom10081166 (PMC7465109; doi:10.3390/biom10081166)
Supplement: Supplementary file 1 [file biomolecules-10-01166-s001.pdf]

Discovery of Sulfated Small Molecule Inhibitors of Matrix Metalloproteinase-8

Shravan Morla [1,2†] and Umesh R. Desai [1,2*]

[1] Department of Medicinal Chemistry, Virginia Commonwealth University, 23298 Richmond, VA, USA; smorla@scripps.edu
[2] Institute for Structural Biology, Drug Discovery and Development, Virginia Commonwealth University, 23219 Richmond, VA, USA
* Correspondence: urdesai@vcu.edu; Tel: 804-828-7575; Fax: 804-827-3664.
† Present Address: Department of Molecular Medicine, The Scripps Research Institute, 92037 La Jolla, CA, USA.

Table of Contents

No.	Title	Pg.
2	**Table S1:** General structure of MMP-8 inhibitors developed to date.	2
3	**Table S2:** List of interactions made by NSGMs with MMP-8.	3
4	**Figure S1:** Comparison of the mode of binding of **38** and **43** on MMP-8.	4
5	**Figure S2:** Mode of binding of the top five NSGM inhibitors (**26**, **38**, **40**, **41**, **42**) of MMP-<br>8 (shown in ESP map).	5
6	**References**	6

Table S1: General structure of MMP-8 inhibitors developed to date.

Compound class	Zinc-binding groups	General structure
Peptide hydroxamates [1]	Hydroxamate	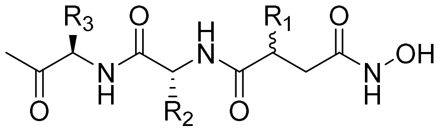
Peptide phosphonates [2-7]	Phosphonate	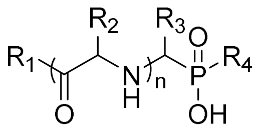
Peptide thiolates [8]	Thiolate	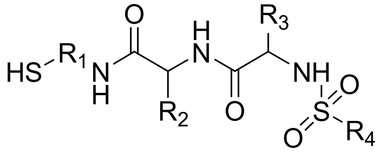
Sulfonamide phosphates [9]	Phosphonate	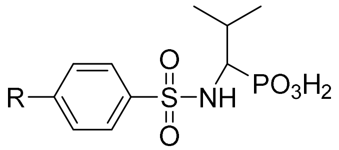
Dihydropyrazothiazoles [10]	-	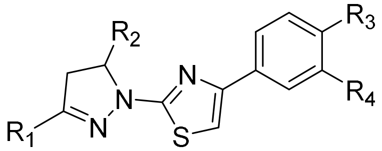
Thienopyrimidinones [11]	-	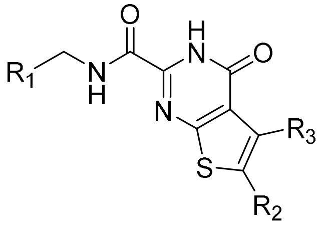

Table S2: List of interactions made by NSGMs with MMP-8.

NSGM	Hydrogen & ionic interactions	Non-bonded interactions
26	H197, H201, H207, S151, Zn^{+2}	E198, Q165, A163, F164, I170, D149, N150, I159, H162, A161
38	L160, A163, L193, L214, P217, A220, R222	V194, N218, G158, L160, I159, S151, F164, A163, H162, H207, H197, A161, E198, Y216
40	H197, H201, H207, Zn^{+2}, Q165, N150, E198	P217, A163, G171, F164, N150, N85
41	E198, H197, H201, H207, Zn^{+2}, H162, S151	I159, E198, P152, F164, A163
42	E198, H197, H201, H207, Zn^{+2}, N157	P217, L160, V194, Y219, H197, E198, H162, I159, N218, G158

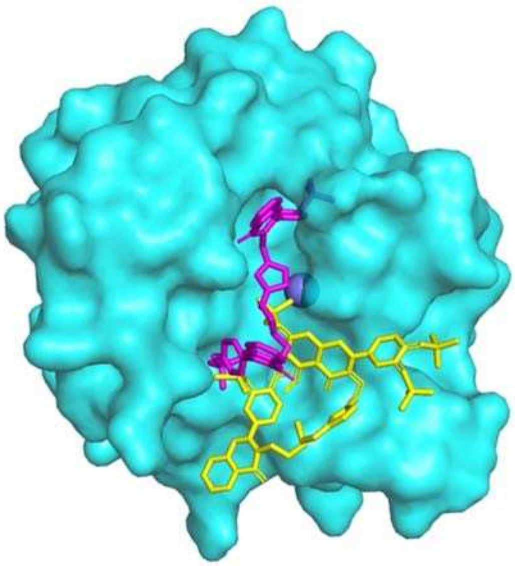

Figure S1: Comparison of the mode of binding of **38** and **43** on MMP-8. The bulk of tetra-sulfated quercetin does not allow its placement in the S_1' pocket, resulting in loss of inhibition of heterodimeric NSGMs **43**– **46**.

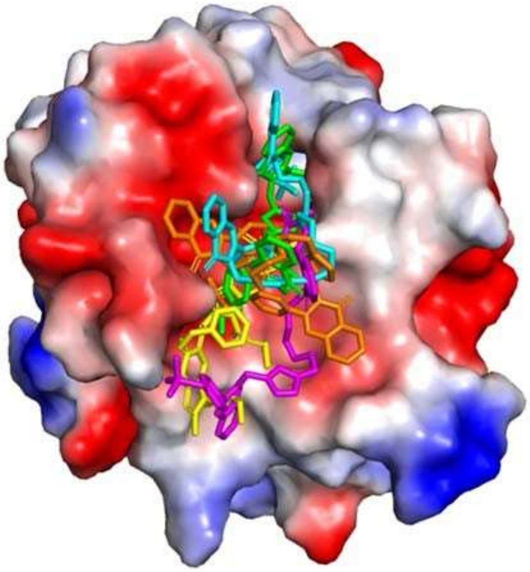

Figure S2: Mode of binding of the top five NSGM inhibitors (**26**, yellow; **38**, green; **40**, magenta; **41**, orange; **42**, cyan – in sticks) of MMP-8 (shown in ESP map).

References:

1. Uttamchandani, M.; Wang, J.; Li, J.; Hu, M.; Sun, H.; Chen, K.; Liu, K.; Yao, S. Q., Inhibitor Fingerprinting of Matrix Metalloproteases Using a Combinatorial Peptide Hydroxamate Library. *J Am Chem Soc* **2007,** *129* (25), 7848-7858.
2. Bhowmick, M.; Tokmina-Roszyk, D.; Onwuha-Ekpete, L.; Harmon, K.; Robichaud, T.; Fuerst, R.; Stawikowska, R.; Steffensen, B.; Roush, W.; Wong, H. R.; Fields, G. B., Second Generation Triple-Helical Peptide Inhibitors of Matrix Metalloproteinases. *J Med Chem* **2017,** *60* (9), 3814-3827.
3. Bianchini, G.; Aschi, M.; Cavicchio, G.; Crucianelli, M.; Preziuso, S.; Gallina, C.; Nastari, A.; Gavuzzo, E.; Mazza, F., Design, modelling, synthesis and biological evaluation of peptidomimetic phosphinates as inhibitors of matrix metalloproteinases MMP-2 and MMP-8. *Bioorg Med Chem* **2005,** *13* (15), 4740-9.
4. Vassiliou, S.; Mucha, A.; Cuniasse, P.; Georgiadis, D.; Lucet-Levannier, K.; Beau, F.; Kannan, r.; Murphy, G.; Knäuper, V.; Rio, M. C.; Basset, P.; Yiotakis, A.; Dive, V., Phosphinic Pseudo- Tripeptides as Potent Inhibitors of Matrix Metalloproteinases: A Structure-Activity Study. *J Med Chem* **1999,** *42* (14), 2610-2620.
5. Mookhtiar, K. A.; Marlowe, C. K.; Bartlett, P. A.; Van Wart, H. E., Phosphonamidate Inhibitors of Human Neutrophil Collagenase. *Biochemistry* **1987,** *27* (7), 1962-1965.
6. Matziari, M.; Georgiadis, D.; Dive, V.; Yiotakis, A., Convenient Synthesis and Diversification of Dehydroalaninyl Phosphinic Peptide Analogues. *Org Lett* **2001,** *3* (5), 659-662.
7. Agamennone, M.; Campestre, C.; Preziuso, S.; Consalvi, V.; Crucianelli, M.; Mazza, F.; Politi, V.; Ragno, R.; Tortorella, P.; Gallina, C., Synthesis and evaluation of new tripeptide phosphonate inhibitors of MMP-8 and MMP-2. *Eur J Med Chem* **2005,** *40* (3), 271-9.
8. Scozzafava, A.; Supuran, C. T., Protease Inhibitors: Synthesis of Matrix Metalloproteinase and Bacterial Collagenase Inhibitors Incorporating 5-Amino-2- mercapto-1,3,4-thiadiazole Zinc Binding Functions. *Bioorg Med Chem Lett* **2002,** *12* (19), 2667-2672.
9. Pochetti, G.; Gavuzzo, E.; Campestre, C.; Agamennone, M.; Tortorella, P.; Consalvi, V.; Gallina, C.; Hiller, O.; Tschesche, H.; Tucker, P. A.; Mazza, F., Structural Insight Into the Stereoselective Inhibition of MMP-8 by Enantiomeric Sulfonamide Phosphonates. *J Med Chem* **2006,** *49* (3), 923- 931.
10. Wang, Z. C.; Shen, F. Q.; Yang, M. R.; You, L. X.; Chen, L. Z.; Zhu, H. L.; Lu, Y. D.; Kong, F. L.; Wang, M. H., Dihydropyrazothiazole derivatives as potential MMP-2/MMP-8 inhibitors for cancer therapy. *Bioorg Med Chem Lett* **2018,** *28* (23-24), 3816-3821.
11. Pochetti, G.; Montanari, R.; Gege, C.; Chevrier, C.; Taveras, A. G.; Mazza, F., Extra Binding Region Induced by Non-Zinc Chelating Inhibitors Into the S1' Subsite of Matrix Metalloproteinase 8 (MMP-8). *J Med Chem* **2009,** *52* (4), 1040-1049.
